# The Impact of Different Classification Criteria Sets on the Estimated Prevalence and Associated Risk Factors of Diastolic Dysfunction in Rheumatoid Arthritis

**DOI:** 10.1155/2017/2323410

**Published:** 2017-12-04

**Authors:** Lebogang Mokotedi, Sulé Gunter, Chanel Robinson, Gavin R. Norton, Angela J. Woodiwiss, Linda Tsang, Patrick H. Dessein, Aletta M. E. Millen

**Affiliations:** ^1^Cardiovascular Pathophysiology and Genomics Research Unit, School of Physiology, Faculty of Health Sciences, University of the Witwatersrand, Johannesburg, South Africa; ^2^Rheumatology Division, Universitair Ziekenhuis Brussel (UZB), Vrije Universiteit Brussel (VUB), Brussels, Belgium

## Abstract

This study compared the estimated prevalence and potential determinants of left ventricular (LV) diastolic dysfunction upon applying different classification criteria in rheumatoid arthritis (RA). LV diastolic function was assessed echocardiographically by pulsed Doppler (*E*/*A*), tissue Doppler (*E*/*e*′, lateral and septal *e*′), and left atrial volume index in 176 RA patients. Relationships of traditional cardiovascular risk factors and RA characteristics with LV diastolic function and dysfunction according to previous and current criteria were determined in multivariate regression models. Waist-hip ratio was associated with *E*/*A* (standardised *β* (SE) = −0.28 ± 0.09, *p* = 0.0002) and lateral *e*′ (standardised *β* (SE) = 0.26 ± 0.09, *p* = 0.01); low diastolic blood pressure was related to *E*/*e*′ (standardised *β* (SE) = −0.16 ± 0.08, *p* = 0.04). Diastolic dysfunction prevalence differed upon applying previous (59%) compared to current (22%) criteria (*p* < 0.0001). One SD increase in waist-hip ratio was associated with diastolic dysfunction when applying current criteria (OR = 2.61 (95% CI = 1.51–4.52), *p* = 0.0006), whereas one SD increase in diastolic blood pressure was inversely related to diastolic dysfunction upon using previous criteria (OR = 0.57 (95% CI = 0.40–0.81), *p* = 0.002). In conclusion, application of current and previous diastolic dysfunction criteria markedly alters the prevalence and risk factors associated with diastolic dysfunction in RA.

## 1. Introduction

Rheumatoid arthritis (RA) increases the risk of heart failure 2-fold [1]. Heart failure accounts for 13% of the mortality in RA [2]. Among heart failure patients, those with RA experience less frequently typical symptoms and signs of heart failure and are more likely to have a preserved ejection fraction (EF) [3, 4]. Left ventricular (LV) diastolic dysfunction is the most common cause of heart failure with preserved ejection fraction and is associated with adverse cardiovascular outcomes including incident heart failure [5, 6]. Metabolic risk factor driven inflammation is strongly implicated in diastolic dysfunction [7]. RA patients experience interrelated high-grade inflammation and increased metabolic risk [8].

In RA, traditional cardiovascular risk factors and coronary artery disease do not fully explain all changes in diastolic dysfunction [9]. In this regard, disease duration [10–21] and circulating concentrations of the inflammatory markers interleukin-6 [17] and tumour necrosis factor-*α* [19] have been associated with diastolic dysfunction in patients with RA. Patients with RA also experience increased left ventricular mass [22], which is associated with diastolic dysfunction. Nevertheless, most previous studies on diastolic dysfunction in RA had small sample sizes [10–14, 19, 23] and traditional cardiovascular risk factors were consistently adjusted for in only two of these [17, 18]. The relative impact of traditional cardiovascular risk factors and disease characteristics beyond structural changes on diastolic dysfunction and its different components in RA remains uncertain.

Diastolic dysfunction is most frequently assessed by echocardiography. Importantly, in the present context, several RA investigations focused on the pulsed Doppler determined early filling velocity (*E*) to atrial contraction velocity (*A*) (*E*/*A*) ratio as a parameter of diastolic function [11, 14, 19–21, 24]. However, measures that require tissue Doppler imaging including particularly the *E*/early peak velocity during diastole at the mitral annulus (*e*′) (*E*/*e*′) ratio were proven to more reliably represent diastolic function as assessed by invasive pressure-volume analysis [25]. In 2003, a criteria set for classification of diastolic dysfunction which included both pulsed and tissue Doppler determined parameters was reported by Redfield and colleagues [26]. Upon applying the respective criteria set, Liang and colleagues documented increased prevalence of diastolic dysfunction among RA patients in a community-based study in 2010 [17]. In 2016, Nagueh and colleagues reported new recommendations for the evaluation of diastolic dysfunction. The *E*/*A* ratio was no longer considered in patients with an ejection fraction of ≥50% and a relatively high cut-off value for raised *E*/*e*′ of 14 cm/sec was proposed [27]. Whether this changed approach can influence the identification of RA patients who are at risk of developing heart failure with preserved ejection fraction is unknown.

In this study, we determined (1) independent relationships of traditional cardiovascular risk factors and disease characteristics with pulsed and tissue Doppler measures of diastolic function and (2) whether application of different diastolic dysfunction classification criteria sets impacts on the estimated prevalence and associated risk factors of diastolic dysfunction in patients with RA.

## 2. Materials and Methods

### 2.1. Patients

We recruited 186 patients that met the 2010 American College of Rheumatology/European League against Rheumatism criteria for RA [28] at the Milpark Hospital in Johannesburg, South Africa. Of these, 176 patients (117 white, 29 Asian, 22 black, and 8 of mixed ancestry) in whom high-quality echocardiographic measures were obtained and who did not have heart failure were included in the study. This investigation was performed in line with the principles of the Helsinki Declaration and approval was obtained from the University of Witwatersrand Human (Medical) Research Ethics Committee (approval number: M06-07-33; protocol number: M120562) in Johannesburg, South Africa. All participants gave written informed consent.

### 2.2. Baseline Characteristics

Baseline characteristics were assessed using standard approaches as previously reported [29]. Demographic characteristics, lifestyle factors, and anthropometric variables were recorded. Systolic blood pressure and diastolic blood pressure (DBP) were measured and hypertension was identified in the presence of systolic blood pressure ≥ 140 mm Hg, diastolic blood pressure ≥ 90 mm Hg, or/and use of antihypertensive agents. Standard laboratory blood tests of renal and liver function, lipids, glucose, haematological variables, and erythrocyte sedimentation rate (ESR) were performed. Dyslipidemia was diagnosed when the cholesterol/high-density lipoprotein (HDL) ratio was >4 [30] or/and lipid-lowering agents were employed. Diabetes was identified when the fasting plasma glucose was ≥7 mmol/l or/and glucose-lowering agents were used.

RA disease duration, rheumatoid factor, and anti-citrullinated peptide antibody (ACPA) status were assessed. Disease activity was estimated by the disease activity score in 28 joints (DAS28) and the clinical disease activity index (CDAI) [31]. Current or previously identified (hospital record review) extra-articular manifestations [29] were recorded. Physical impairment was evaluated by the Stanford Health Assessment Questionnaire disability index (HAQ-DI). C-reactive protein (CRP) concentrations were determined using immunoturbidimetric methods. Renal function was assessed by the Chronic Kidney Disease Epidemiology Collaboration (CKD-EPI) estimated glomerular filtration rate (eGFR) [32]. Insulin resistance was estimated by the homeostasis model assessment for insulin resistance (HOMA-IR) [33].

### 2.3. Echocardiography

Echocardiography was performed using SonoSite's M-Turbo ultrasound (SonoSite® Inc., Bothell, WA, USA) with the patient in the partial left decubitus position according to the American Society of Echocardiography convention [34]. LV dimensions were determined by measuring the left ventricular internal end-diastolic and end-systolic diameters and wall thickness (interventricular septal and posterior wall thickness) in the parasternal long axis view by two-dimensional directed M-mode echocardiography. Left ventricular end-diastolic and systolic volumes were assessed using the Teichholz method [35]. LV ejection fraction was calculated as [(LV end-diastolic volume − LV end-systolic volume)/LV end-diastolic volume] × 100. Left ventricular mass was determined using a standard formula [36] and indexed to body surface area (LVMI). LV relative wall thickness (RWT) was calculated as (LV diastolic posterior wall thickness × 2)/LV end-diastolic diameter [37]. Left ventricular hypertrophy was identified when left ventricular mass index was >95 g/m^2^ for women and >115 g/m^2^ for men [36].

Left ventricular diastolic function was assessed using pulsed Doppler, tissue Doppler imaging, and left atrial volume [27]. Transmitral flow patterns were recorded at the mitral valve leaflet tips using pulsed Doppler in the apical four-chamber view. From the mitral valve inflow velocity curve, the early (*E*) and late (atrial-*A*) diastolic wave were measured and the ratio of early and late diastolic filling (*E*/*A*) was calculated. To perform tissue Doppler imaging, the velocity of myocardial tissue lengthening was measured by placing the curser at the septal and lateral corners of the mitral annulus in the apical four-chamber view. To determine diastolic function using tissue Doppler imaging, the peak velocities during early (*e*′) and late (*a*′) diastole were recorded. Because mitral *E* is dependent on ventricular relaxation as well as left atrial driving forces (pressures), while *e*′ is dependent on relaxation alone, *E*/*e*′ is considered to be an index of left ventricular filling pressures. The *E*/*e*′ ratio was calculated as mitral *E*/the average of septal and lateral *e*′. Left atrial area was determined using planimetry at end-systole in the apical four-chamber or two-chamber view from which left atrial volume was calculated by the area-length method and was indexed to body surface area (LAVI). The presence of diastolic dysfunction was determined using previously [26] and currently reported approaches [27]. Briefly, according to previous approaches, mild diastolic dysfunction was diagnosed when *E*/*A* was <0.75 and *E*/*e*′ < 10, moderate diastolic dysfunction was diagnosed when 0.75 ≤ *E*/*A* < 1.5 and *E*/*e*′ ≥ 10, and severe diastolic dysfunction was diagnosed when *E*/*A* was ≥1.5 and *E*/*e*′ ≥ 10 [26]. According to the current recommendations, in patients with an ejection fraction ≥ 50%, diastolic dysfunction was identified when at least 2 of the following criteria were met: *E*/*e*′ > 14, lateral *e*′ < 10 cm/sec or septal *e*′ < 7 cm/sec, and left atrial volume index > 34 ml/m^2^; in those with an ejection fraction < 50%, mild diastolic dysfunction was diagnosed when *E*/*A* was <0.8, severe diastolic dysfunction was diagnosed when *E*/*A* was >2, and moderate diastolic dysfunction was diagnosed when *E*/*A* was >0.8 and <2, *E*/*e*′ > 14, and left atrial volume index > 34 ml/m^2^ [27].

Echocardiography was performed by two experienced operators that were blinded to the clinical data and cardiovascular disease risk factor profiles of the patients. All echocardiographic images were reviewed by a single experienced operator. Intra- and interobserver studies were conducted on 20 individuals. For the interobserver variability, Pearson's correlation coefficients for LV end-diastolic diameter, septal wall thickness, posterior wall thickness, *E*, *A*, and *e*′ were 0.71, 0.84, 0.81, 0.95, 0.92, and 0.92, respectively (*p* < 0.0001 for all) and the coefficients of variation for LV end-diastolic diameter, septal wall thickness, posterior wall thickness, *E*, *A*, and *e*′ were 2.7%, 2.9%, 2.6%, 1.9%, 3.9%, and 4.2%, respectively. There were no significant differences between the measures on unpaired *t*-tests (*p* > 0.2 for all). For the intraobserver variability, Pearson's correlations for repeat measures ranged between 0.74 and 0.98 for one observer and between 0.82 and 0.94 for the other observer for all the aforementioned measures (all *p* < 0.001). The respective coefficients of variation ranged between 1.4% and 4.7% for one observer and between 1.2% and 5.4% for the other observer for all the aforementioned measures. There were no differences in the repeat measures on paired *t*-tests for either observer (*p* > 0.2 for all).

### 2.4. Statistical Analysis

Database management and statistical analyses were performed using SAS software, version 9.4 (SAS Institute Inc., Cary, North Carolina, USA). Descriptive data are presented as mean (SD), median (interquartile range (IQR)), or proportions for normally distributed, nonnormally distributed, and categorical variables, respectively. Nonnormally distributed variables were logarithmically transformed in order to improve skewness and kurtosis for statistical analysis. Potential relationships of baseline characteristics including traditional cardiovascular risk factors and RA features with markers of diastolic function (as well as LV mass parameters) were first assessed in age-, sex-, and race-adjusted linear regression models. The independent associations of baseline characteristics with diastolic function parameters and diastolic dysfunction according to previous and current diastolic dysfunction classification criteria sets were subsequently determined in linear and backward logistic regression models, respectively. As LV mass and concentric hypertrophy are strongly associated with diastolic function, the associations of LV mass index and relative wall thickness with diastolic function parameters were determined. LV mass index was associated with *E*/*A* ratio (*r* = −0.30; *p* < 0.0001), lateral *e*′ (*r* = −0.30; *p* < 0.0001), septal *e*′ (*r* = −0.23; *p* = 0.002), and LAVI (*r* = 0.19; *p* = 0.009); relative wall thickness was not associated with any LV diastolic function parameters. In age-, sex-, and race-adjusted analysis, neither LV mass index nor relative wall thickness was associated with diastolic dysfunction parameters (data not shown). Nevertheless, as LV mass index was more strongly and consistently associated with diastolic function parameters than relative wall thickness in univariate analysis, LV mass index was forced in additional regression models with diastolic dysfunction as dependent variable. Dyslipidemia, smoking, and diabetes were added as independent variables into final models on diastolic dysfunction among all RA patients. As the impact of risk factors on cardiovascular disease can differ in women compared to men, the independent associations of baseline characteristics with diastolic dysfunction were also assessed in a sensitivity analysis among women. Lastly, sensitivity analysis was performed in patients without established cardiovascular disease (*n* = 9) and in those with an ejection fraction of ≥50% (isolated diastolic dysfunction). Significance was set at *p* ≤ 0.05.

## 3. Results

### 3.1. Patient Characteristics

Table 1 gives the recorded characteristics. Mean (SD) age and median (IRQ) disease duration were 58.4 (12.4) years and 14.5 (8.9–22) years, respectively. Of the participants, 40% had hypertension, 49% dyslipidemia, and 5% diabetes. All patients with hypertension received antihypertensive agents. Disease activity was overall well controlled with a median (IQR) CDAI of 6 (1–13) and mean (SD) DAS28 of 2.8 (1.7); 73.7% of the patients tested rheumatoid factor-positive and 69.5% ACPA-positive; 22% experienced extra-articular manifestations.

The mean (SD) ejection fraction was 58.7 (13.2) %; 21% of participants had an ejection fraction of <50% and 19.3% left ventricular hypertrophy. The *E*/*e*′ ratio was ≥10 and >14 in 58% and 20% of participants, respectively. Upon applying previously reported criteria [26], 58.6% of the patients were classified as having diastolic dysfunction and none had indeterminate diastolic function; when the recently reported criteria [27] were applied, 22% had diastolic dysfunction and 23% indeterminate diastolic function; 21.6% of the participants were classified with diastolic dysfunction according to both criteria sets, 37.4% met the previous but not recent criteria, and 1.2% met the recent but not previous criteria.

The estimated proportion of RA persons with diastolic dysfunction differed significantly when applying previous [26] compared to current criteria [27] (*p* < 0.0001).

### 3.2. Relationships of Traditional Risk Factors and RA Characteristics with Indices of LV Diastolic Function and LV Geometry

The significant age-, sex-, and race-adjusted associations of baseline characteristics with markers of diastolic function and LV geometry are shown in Supplementary Table  1 in Supplementary Material available online at https://doi.org/10.1155/2017/2323410. Age was associated with all markers of diastolic function (*p* < 0.01 for all) and LV mass index (partial *r* = 0.36; *p* < 0.0001) but not relative wall thickness (*p* = 0.4). Female sex (partial *r* = 0.16; *p* = 0.03) and heart rate (partial *r* = −0.29; *p* < 0.001) were associated with *E*/*A*. Estimated glomerular filtration rate was directly related to *E*/*A* (partial *r* = 0.22; *p* = 0.01), lateral *e*′ (partial *r* = 0.27; *p* = 0.002), and septal *e*′ (partial *r* = 0.27; *p* = 0.001) and inversely associated with left atrial volume index (partial *r* = −0.17; *p* = 0.04) and LV mass index (partial *r* = −0.24; *p* = 0.005). Waist-hip ratio (WHR) comprised the adiposity marker that was most consistently associated diastolic function marker and was therefore included in subsequent analysis. Waist-hip ratio was associated with low *E*/*A* (partial *r* = −0.24; *p* = 0.003) and decreased lateral *e*′ (partial *r* = −0.34; *p* < 0.001) and with increased LAVI (partial *r* = 0.27; *p* = 0.0005) and LV mass index (partial *r* = 0.19; *p* = 0.02). Diastolic blood pressure was related to *E*/*e*′ (partial *r* = −0.16; *p* = 0.04) and lateral *e*′ (partial *r* = 0.19; *p* = 0.02) and hypertension was related to LAVI (partial *r* = 0.27; *p* = 0.0003). Triglycerides were inversely related to lateral *e*′ (partial *r* = −0.2; *p* = 0.01) and directly related to LAVI (partial *r* = 0.25; *p* = 0.001). HOMA-IR was inversely associated with *E*/*A* (partial *r* = −0.17; *p* = 0.04) and lateral *e*′ (partial *r* = −0.16; *p* = 0.05) and directly with LVAI (partial *r* = 0.18; *p* = 0.03).

Among RA characteristics, disease duration was most consistently associated with diastolic function markers including *E*/*A* (partial *r* = −0.25; *p* = 0.0001), lateral *e*′ (partial *r* = −0.28; *p* = 0.0002), left atrial volume index (partial *r* = 0.21; *p* = 0.007), and LV mass index (partial *r* = 0.20; *p* = 0.01). DAS 28 (partial *r* = −0.17; *p* = 0.03), erythrocyte sedimentation rate (partial *r* = −0.20; *p* = 0.01), and C-reactive protein (partial *r* = −0.16; *p* = 0.04) were related to *E*/*A*. ACPA status (partial *r* = 0.18; *p* = 0.02) and white cell count (partial *r* = 0.19; *p* = 0.01) related to relative wall thickness. The presence of extra-articular manifestations was associated with LAVI (partial *r* = 0.16; *p* = 0.04).

Baseline characteristics that were not significantly related to diastolic function parameters are given in Supplementary Table  2.

Clinical characteristics that were associated with diastolic function markers in the previous analysis were then entered into separate linear regression models with diastolic function markers as dependent variables.  Table 2 gives the independent relationships of traditional cardiovascular risk factors and RA characteristics with diastolic function markers. The results are expressed as standardised *β* (SE) to allow for comparison of the contributions of the different baseline recorded characteristics to the variation in diastolic function. Age at disease onset (*β* (SE) = −0.31 (0.11), *p* = 0.0006), race (*β* (SE) = 0.19 (0.09), *p* = 0.02), heart rate (*β* (SE) = −0.21 (0.08), *p* = 0.01), and waist-hip ratio (*β* (SE) = −0.28 (0.09), *p* = 0.002) remained significantly associated with *E*/*A*. Age (*β* (SE) = 0.19 (0.08), *p* = 0.01) and diastolic blood pressure (*β* (SE) = −0.16 (0.08), *p* = 0.04) were associated with *E*/*e*′. Age at disease onset (*β* (SE) = −0.34 (0.11), *p* = 0.002) and waist-hip ratio (*β* (SE) = −0.26 (0.09), *p* = 0.01) were associated with lateral *e*′. Age at disease onset (*β* (SE) = −0.46 (0.10), *p* < 0.0001), disease duration (*β* (SE) = −0.35 (0.09), *p* = 0.0003), and azathioprine use (*β* (SE) = −0.24 (0.08), *p* = 0.005) were associated with septal *e*′. Race (*β* (SE) = −0.22 (0.09), *p* = 0.01), waist-hip ratio (*β* (SE) = 0.27 (0.11), *p* = 0.01), and triglyceride concentrations (*β* (SE) = 0.20 (0.09), *p* = 0.03) were associated with left atrial volume index.

### 3.3. Independent Relationships of Traditional Risk Factors and RA Characteristics with LV Diastolic Dysfunction according to Previous and Current Criteria Sets

Baseline characteristics that remained associated with diastolic function markers in Table 2 were subsequently entered into backward logistic regression models with diastolic dysfunction according to previously [26] or currently reported [27] criteria sets as dependent variables. As shown in Table 3, age at disease onset (OR = 2.28 (95% CI = 1.22–4.25), *p* = 0.01) and the waist-hip ratio (OR = 2.61 (95% CI = 1.51–4.52), *p* = 0.0006) were associated with diastolic dysfunction upon using the current criteria; these relationships were materially unaltered after adding LV mass index (*p* = 0.005 and *p* = 0.0004, resp.) as an additional potential confounder to the model. Upon applying the previous criteria, only a low diastolic blood pressure (OR = 0.57 (95% CI = 0.40–0.81), *p* = 0.002) was associated with diastolic dysfunction; low diastolic blood pressure remained independently associated with diastolic dysfunction (according to previous criteria) when LV mass index (*p* = 0.003) was added to the model. When adding smoking, dyslipidemia, and diabetes to the models, age at disease onset (OR = 2.27 (95% CI = 1.18–4.37), *p* = 0.01) and waist-hip ratio (OR = 2.44 (95% CI = 1.39–4.27), *p* = 0.002) remained associated with diastolic dysfunction according to previous criteria and low diastolic blood pressure (OR = 0.56 (95% CI = 0.39–0.81), *p* = 0.002) remained related to diastolic dysfunction according to recent criteria. After exclusion of patients with established cardiovascular disease (*n* = 9), the results were materially unaltered (data not shown).

As given in Table 4, in sensitivity analysis among women, waist-hip ratio (OR = 3.49 (95% CI = 1.79–6.82), *p* = 0.0002) was associated with diastolic dysfunction according to current criteria and a low diastolic blood pressure (OR = 0.62 (95% CI = 0.42–0.92), *p* = 0.02) was related to diastolic dysfunction according to previous criteria. When adding LV mass index to the models, these associations remained significant. Sensitivity analysis was not performed in men due to the small numbers involved (*n* = 32).

In Table 5, a sensitivity analysis among patients with an ejection fraction of ≥50% was performed. Age at disease onset was associated with diastolic dysfunction according to the current criteria (OR = 2.45 (95% CI = 1.20–5.02), *p* = 0.01). When LV mass index was added to the model, age at disease onset remained associated (*p* = 0.01) with diastolic dysfunction according to the current criteria. Additionally, waist-hip ratio (OR = 1.99 (95% CI = 1.04–3.80), *p* = 0.03) and disease duration (OR = 1.99 (95% CI = 1.01–3.93), *p* = 0.05) were related to diastolic dysfunction according to the current criteria. A low diastolic blood pressure was significantly associated with diastolic dysfunction according to previous criteria before (OR = 0.59 (95% CI = 0.40–0.89), *p* = 0.01) and after (OR = 0.60 (95% CI = 0.40–0.91), *p* = 0.01) LV mass index was added to the model.

When azathioprine was omitted as independent variable from the models in Tables 3, 4 and 5, the results were unaltered (data not shown).

## 4. Discussion

This study examined the potential contribution of comprehensively assessed traditional cardiovascular risk factors and disease characteristics to LV diastolic function in patients with RA. Our analysis revealed that waist-hip ratio was independently associated with the *E*/*A* ratio, lateral *e*′, and left atrial volume index, whereas low diastolic blood pressure related to the *E*/*e*′ ratio. We also report for the first time that the estimated prevalence of diastolic dysfunction is markedly lower upon application of the recently reported classification criteria by Nagueh and colleagues [27] compared to those previously recommended by Redfield and colleagues [26] (22.0% versus 58.6%) in RA. Moreover, the risk factors associated with diastolic dysfunction differed consistently when previous compared to current classification criteria sets were used.

We found that waist-hip ratio contributed to the variability of *E*/*A* ratio and *e*′ to a similar extent as age at disease onset, and diastolic blood pressure was as strongly related to *E*/*e*′ as age. Adequate LV diastolic function depends on active and passive processes. The active process comprises high energy requiring relaxation [38]. It involves rapid removal of cytosolic calcium into the sarcoplasmic reticulum, thin filament deactivation, and cross-bridging kinetics [38]. The passive process consists of cardiac chamber compliance that is reduced by the expression and cross-linking of collagen in the interstitium and alterations in titin expression within sarcomeres when the ventricle stiffens [39]. In the present study, waist-hip ratio was inversely related to the *E*/*A* ratio and *e*′, and diastolic blood pressure was inversely associated with *E*/*e*′. In this regard, a low *E*/*A* ratio and *e*′ represent impaired relaxation, whereas a large *E*/*e*′ ratio is an index of filling pressure and is more strongly associated with fibrosis as part of myocardial stiffness [40, 41]. Taken together, our results indicate that excess central adiposity may impair LV relaxation, whereas low diastolic blood pressure may contribute to myocardial fibrosis and a stiffened ventricle in RA.

The question arises as to why there is a 2- to 3-fold difference in estimated diastolic dysfunction prevalence as well as a consistent disparity in associated risk factors by applied classification criteria in RA. Both criteria sets include pulsed and tissue Doppler measurements. However, in contrast to previously reported criteria, the *E*/*A* ratio is not included as a criterion among patients with an ejection fraction ≥ 50% in the recent recommendations. Additionally, the *E*/*e*′ ratio is considered as a criterion when its value is ≥10 cm/sec in the previous compared to >14 cm/sec in the recent recommendations. In our study, waist-hip ratio was related to the *E*/*A* ratio and diastolic dysfunction according to the recent but not previous criteria. On the other hand, diastolic blood pressure was associated with the *E*/*e*′ ratio and diastolic dysfunction according to the previous but not current criteria. Moreover, 20% of our patients had *E*/*e*′ ratio of >14 and 58% experienced *E*/*e*′ ratio of ≥10. These findings suggest that the reduced sensitivity in classifying patients as having diastolic dysfunction by the recent criteria set is at least partly due to the high threshold that is used upon considering an increase in *E*/*e*′ ratio. Our results indicate the need for further studies aimed at determining adequate heart failure risk stratification in RA. Interestingly, a recent population study similarly reported marked differences in the prevalence and predictors of diastolic dysfunction according to different classification criteria [42].

In the present study, 1 SD increase in diastolic blood pressure reduced the odds ratio for diastolic dysfunction according to previous criteria to 0.57, whereas 1 SD increase in waist-hip ratio increased the odds ratio for diastolic dysfunction according to current criteria 2.61-fold in patients with RA. These relationships were independent of LV mass index and reproduced in sensitivity analyses among women and patients without established cardiovascular disease as well as those with isolated diastolic dysfunction. In aged subjects, low diastolic blood pressure forms part of an increased pulse pressure, which is mediated by increased arterial stiffness and wave reflection [43]. Neither systolic blood pressure nor pulse pressure was associated with diastolic function markers or diastolic dysfunction classification in this study. In this regard, low diastolic blood pressure in aged subjects was found to enhance LV stiffening by reducing coronary perfusion during diastole [44]. In the non-RA population, excess central adiposity was shown to play an important role in the development of diastolic dysfunction [45–49]. In patients with RA, central adiposity is associated with systemic inflammation and metabolic risk factors [50]. Also, the waist-hip ratio is related to atherosclerosis in RA and this association is explained by metabolic risk factors [51]. Our current findings suggest that low diastolic blood pressure may need to be considered in cardiovascular risk evaluation and further support the role of central obesity in cardiovascular disease risk among RA patients.

An association between RA duration and diastolic function parameters or/and dysfunction according to reported criteria was previously reported in some [10–16] but not all studies [20, 21, 23, 52]. In the present investigation, RA duration was related to several diastolic function parameters in age-, sex-, and race-adjusted analysis. However, in fully adjusted models, RA duration remained associated with septal *e*′ only. RA duration was associated with diastolic dysfunction according to the new criteria only in those with an ejection fraction ≥ 50% and after adjustment for LV mass index. DAS28, erythrocyte sedimentation rate, and C-reactive protein concentrations were associated with the *E*/*A* ratio in age-, sex-, and race-adjusted analysis but not in fully adjusted models.

Our study design was cross-sectional, thereby precluding drawing conclusions on cause and effect relationships. Our findings require further evaluation in future longitudinal studies. We did not record echocardiographically determined tricuspid regurgitation velocity. The left atrial volume index was remarkably low in this RA study. Upon application of diastolic classification criteria, we used left atrial volume index threshold values as given in recent recommendations that are largely based on data obtained in American populations [27]. In this regard, Chahal and colleagues [53] reported that the left atrial volume index differs substantially across population groups. Also, previous studies performed in South Africa have reported similar low left atrial volume indices as found in our present study [54, 55]. We may therefore have underestimated the actual prevalence of diastolic dysfunction according to the new criteria. Further studies are needed to determine optimal left atrial volume index threshold values in the classification of diastolic dysfunction in the South African context. Lastly, the *E*/*A* ratio may be affected by pseudonormalization [26], which could have impacted our results. Nevertheless, whereas the *E*/*A* ratio is not included among the new diastolic dysfunction classification criteria upon their application in persons without systolic dysfunction [27], our results were materially unaltered in a sensitivity analysis among RA patients with isolated diastolic dysfunction.

In conclusion, central adiposity and low diastolic blood pressure are independently associated with diastolic function in patients with RA. The application of different reported criteria markedly influences the estimated prevalence and associated risk factors of diastolic dysfunction in RA. Future studies are urgently needed in order to further improve our understanding of heart failure risk and its stratification among patients with RA.

## Supplementary Material

Supplementary Table 1: Traditional risk factors and RA characteristics associated with diastolic function and left ventricular geometry. Supplemental Table 2: Non-significant associations of traditional risk factors and RA characteristics with markers of diastolic function and left ventricular geometry.

## Figures and Tables

**Table 1:** Recorded characteristics in 176 patients with RA.

*Demographic characteristics*	
Age at study time, years	58.4 (12.4)
Age at disease onset, years	42.3 (14.1)
Female sex, %	81.6
*Lifestyle factors*	
Exercise, %	32.4
Alcohol use, %	31.8
Currently smoking, %	10.1
*Anthropometry*	
Body mass index, kg/m^2^	26.7 (5.3)
Waist circumference, cm	91 (13)
Waist-hip ratio	0.88 (0.09)
*Metabolic risk factors*	
Hypertension, %	40.2
SBP, mm Hg	128 (15)
DBP, mm Hg	81 (8)
Pulse pressure, mm Hg	47 (13)
Total cholesterol, mmol/l	4.5 (1.1)
HDL cholesterol, mmol/l	1.64 (0.47)
LDL cholesterol, mmol/l	2.42 (0.88)
Triglycerides, mmol/l	0.95 (0.7–1.3)
Cholesterol-HDL ratio	2.7 (2.3–3.3)
Dyslipidemia, %	49.2
Diabetes, %	5.0
Glucose, mmol/l	4.8 (4.5–5.1)
HOMA-IR	1.4 (1.0–1.9)
*Cardiovascular agents, %*	
Antihypertensives	40.2
Statins	41.9
Ezetimibe	9.5
Oral glucose-lowering agents	1.7
Insulin	1.7
*RA characteristics*	
RA duration, years	14.5 (8.9–22.0)
Rheumatoid factor-positive, %	73.7
ACPA-positive, %	69.5
CDAI	6 (1–13)
DAS 28	2.8 (1.7)
ESR, mm/h	12 (4–26)
CRP, mg/l	3.2 (1.2–8.0)
Leukocytes, *n*/nl	5.5 (4.6–7.1)
Deformed joints, *n*	0 (0–11)
Extra-articular manifestations, %	22
Stanford HAQ-DI	0.38 (0.0–0.88)
*Synthetic DMARD, %*	
Methotrexate	75.4
Chloroquine	48.0
Leflunomide	40.2
Sulphasalazine	15.6
Tetracycline	16.2
Azathioprine	6.7
Current DMARDS, *n*	2.0 (1.1)
*Biological DMARD, %*	
TNF-*α* inhibitors	8.4
Abatacept	2.2
NSAID, %	32.9
Prednisone use, %	2.2
GFR, ml/min/1.73 m^2^	99.3 (87.7–107.6)
Heart rate, beats per minute	72 (12)
Framingham score	3.02 (5.38)
*Echocardiographic measures*	
*E*/*A*	1.01 (0.82–1.32)
*E*/*e*′	11.10 (8.01–13.42)
Lateral *e*′, cm/s	9.60 (7.68–12.20)
Septal *e*′, cm/s	7.94 (6.38–9.64)
Left ventricular mass index, g/m^2^	78.19 (66.11–92.72)
Left ventricular hypertrophy, %	19.3
Left ventricular RWT	0.44 (0.08)
Concentric remodeling, %	55.1
Stroke volume, ml	56.10 (20.21)
Ejection fraction, %	58.66 (13.20)
Depressed ejection fraction, %	21
Left atrial volume, ml	36.28 (11.51)
Left atrial volume index, ml/m^2^	20.47 (5.92)
Increased LAVI, %	2.8
*Classification of DD*	
DD by previous criteria, %	58.6
DD by new criteria, %	22.0

SBP, systolic blood pressure; DBP, diastolic blood pressure; HDL, high-density lipoprotein; LDL, low-density lipoprotein; HOMA-IR, homeostasis model of insulin resistance; ACPA, anti-citrullinated protein antibody; CDAI, clinical disease activity index; DAS28, disease activity score in 28 joints; ESR, erythrocyte sedimentation rate; CRP, C-reactive protein; HAQ-DI, Health Assessment Questionnaire disability index; DMARD, disease-modifying antirheumatic drugs; NSAID, nonsteroidal anti-inflammatory drugs; GFR, glomerular filtration rate; RWT, relative wall thickness; LAVI, left atrial volume index; DD, diastolic dysfunction.

**Table 2:** Independent relationships of traditional risk factors and RA disease characteristics with markers of diastolic function.

	log⁡*E*/*A*			log⁡*E*/*e*′			log⁡lateral *e*′			log⁡septal *e*′			LAVI		
	Std. *β*	SE	*p*	Std. *β*	SE	*p*	Std. *β*	SE	*p*	Std. *β*	SE	*p*	Std. *β*	SE	*p*
Age				0.19	0.08	0.01									
Age at disease onset	−0.31	0.11	0.006				−0.34	0.11	0.002	−0.46	0.10	<0.0001			
Race	0.19	0.09	0.02										−0.22	0.09	0.01
DBP				−0.16	0.08	0.04									
HR	−0.21	0.08	0.01												
WHR	−0.28	0.09	<0.0001				−0.26	0.09	0.01				0.27	0.11	0.01
Disease duration										−0.35	0.09	0.0003			
Triglycerides													0.20	0.09	0.03
Azathioprine										−0.24	0.08	0.005			

DBP, diastolic blood pressure; HR, heart rate; WHR, waist-hip ratio.

**Table 3:** Independent relationships of 1 SD increase in traditional risk factors and RA disease characteristics with diastolic dysfunction based on new and previous criteria in all patients.

	Diastolic dysfunction by new criteria				Diastolic dysfunction by previous criteria			
	Without LVMI in model		With LVMI in model		Without LVMI in model		With LVMI in model	
	Odds ratio (95% CI)	*p*	Odds ratio (95% CI)	*p*	Odds ratio (95% CI)	*p*	Odds ratio (95% CI)	*p*
Age at disease onset	**2.28 (1.22**–**4.25)**	**0.01**	**2.65 (1.34**–**5.20)**	**0.005**	1.32 (0.84–2.06)	0.23	1.51 (0.95–2.42)	0.08
Sex	3.04 (0.95–9.75)	0.06	2.95 (0.90–9.66)	0.07	1.61 (0.59–4.40)	0.35	1.41 (0.50–3.96)	0.51
Race	1.01 (0.70–1.46)	0.96	0.98 (0.67–1.42)	0.89	1.01 (0.75–1.36)	0.95	1.02 (0.75–1.37)	0.92
Heart rate	0.95 (0.62–1.43)	0.79	0.94 (0.62–1.43)	0.77	1.13 (0.78–1.64)	0.51	1.07 (0.73–1.56)	0.74
Diastolic blood pressure	0.71 (0.46–1.08)	0.11	0.73 (0.48–1.11)	0.14	**0.57 (0.40**–**0.81)**	**0.002**	**0.57 (0.40**–**0.83)**	**0.003**
Waist-hip ratio	**2.61 (1.51**–**4.52)**	**0.0006**	**2.76 (1.58**–**4.85)**	**0.0004**	1.37 (0.89–2.12)	0.16	1.45 (0.93–2.26)	0.10
Disease duration	1.57 (0.90–2.74)	0.12	1.69 (0.95–3.01)	0.07	1.40 (0.93–2.12)	0.10	1.50 (0.98–2.29)	0.06
Azathioprine	1.02 (0.19–5.42)	0.98	0.90 (0.16–4.93)	0.90	1.11 (0.27–4.67)	0.88	0.74 (0.16–3.39)	0.70
Triglycerides	1.00 (0.87–1.16)	0.98	1.00 (0.87–1.16)	0.98	1.07 (0.95–1.21)	0.26	1.08 (0.95–1.22)	0.24
LVMI	—		0.77 (0.47–1.29)	0.32	—		0.73 (0.49–1.11)	0.14

LVMI, left ventricular mass indexed to body surface area.

**Table 4:** Independent relationships of 1 SD increase in traditional risk factors and RA disease characteristics with diastolic dysfunction based on new and previous criteria in women with RA.

	Diastolic dysfunction by new criteria				Diastolic dysfunction by previous criteria			
	Without LVMI in model		With LVMI in model		Without LVMI in model		With LVMI in model	
	Odds ratio (95% CI)	*p*	Odds ratio (95% CI)	*p*	Odds ratio (95% CI)	*p*	Odds ratio (95% CI)	*p*
Age at disease onset	1.80 (0.88–3.71)	0.11	2.01 (0.94–4.33)	0.07	1.26 (0.76–2.10)	0.37	1.44 (0.84–2.47)	0.18
Race	1.00 (0.65–1.56)	0.99	0.96 (0.62–1.49)	0.85	1.30 (0.90–1.88)	0.16	1.34 (0.92–1.97)	0.13
Heart rate	0.96 (0.60–1.55)	0.88	0.98 (0.61–1.58)	0.95	1.02 (0.67–1.56)	0.93	0.97 (0.63–1.50)	0.9
Diastolic blood pressure	0.81 (0.51–1.27)	0.35	0.82 (0.52–1.29)	0.39	**0.62 (0.42**–**0.92)**	**0.02**	**0.62 (0.42**–**0.93)**	**0.02**
Waist-hip ratio	**3.49 (1.79**–**6.82)**	**0.0002**	**3.56 (1.80**–**7.03)**	**0.0003**	1.30 (0.80–2.10)	0.29	1.41 (0.88–2.26)	0.24
Disease duration	1.30 (0.67–2.51)	0.43	1.36 (0.69–2.69)	0.37	1.32 (0.83–2.08)	0.24	1.41 (0.88–2.26)	0.15
Azathioprine	0.83 (0.08–8.23)	0.87	0.79 (0.08–8.25)	0.85	0.78 (0.15–4.12)	0.77	0.43 (0.07–2.59)	0.36
Triglycerides	1.05 (0.88–1.25)	0.61	1.04 (0.87–1.25)	0.64	1.16 (0.97–1.38)	0.11	1.16 (0.97–1.39)	0.1
LVMI	—		0.88 (0.50–1.55)	0.65	—		0.81 (0.51–1.29)	0.38

LVMI, left ventricular mass indexed to body surface area.

**Table 5:** Independent relationships of 1 SD increase in traditional risk factors and RA disease characteristics with diastolic dysfunction based on new and previous criteria in RA patients with isolated diastolic dysfunction (EF ≥ 50%).

	Diastolic dysfunction by new criteria				Diastolic dysfunction by previous criteria			
	Without LVMI in model		With LVMI in model		Without LVMI in model		With LVMI in model	
	Odds ratio (95% CI)	*p*	Odds ratio (95% CI)	*p*	Odds ratio (95% CI)	*p*	Odds ratio (95% CI)	*p*
Age at disease onset	**2.45 (1.20**–**5.02)**	**0.01**	**3.04 (1.36**–**6.81)**	**0.01**	1.35 (0.83–2.20)	0.23	1.47 (0.88–2.45)	0.14
Sex	3.55 (0.74–17.02)	0.11	3.37 (0.70–16.23)	0.13	1.25 (0.37–1.24)	0.72	1.11 (0.32–3.83)	0.87
Race	1.08 (0.71–1.67)	0.71	1.09 (0.71–1.69)	0.70	1.07 (0.77–1.49)	0.69	1.05 (0.76–1.47)	0.76
Heart rate	0.97 (0.59–1.59)	0.90	0.94 (0.57–1.55)	0.82	1.02 (0.67–1.53)	0.94	0.96 (0.63–1.46)	0.84
Diastolic blood pressure	0.72 (0.43–1.20)	0.20	0.71 (0.42–1.20)	0.21	**0.59 (0.40**–**0.89)**	**0.01**	**0.60 (0.40**–**0.91)**	**0.01**
Waist-hip ratio	1.76 (0.94–3.30)	0.07	**1.99 (1.04**–**3.80)**	**0.03**	1.20 (0.73–1.97)	0.48	1.28 (0.77–2.14)	0.34
Disease duration	1.78 (0.93–3.39)	0.08	**1.99 (1.01**–**3.93)**	**0.05**	1.51 (0.95–2.41)	0.08	1.58 (0.98–2.55)	0.06
Azathioprine	0.63 (0.06–6.33)	0.70	0.57 (0.06–5.75)	0.64	0.49 (0.10–2.56)	0.40	0.45 (0.09–2.34)	0.34
Triglycerides	0.99 (0.81–1.22)	0.94	0.99 (0.81–1.23)	0.38	1.05 (0.92–1.20)	0.43	1.06 (0.92–1.21)	0.43
LVMI	—		0.65 (0.35–1.24)	0.19	—		0.73 (0.46–1.15)	0.18

EF, ejection fraction; LVMI, left ventricular mass indexed to body surface area.
