# Supplementary material for: The Impact of Different Classification Criteria Sets on the Estimated Prevalence and Associated Risk Factors of Diastolic Dysfunction in Rheumatoid Arthritis
Source: Int J Rheumatol. 2017 Dec 4;2017:2323410. doi: 10.1155/2017/2323410 (PMC5733615; doi:10.1155/2017/2323410)
Supplement: Supplementary file 1 — Supplementary Table 1: Traditional risk factors and RA characteristics associated with diastolic function and left ventricular geometry. Supplemental Table 2: Non-significant associations of traditional risk factors and RA characteristics with markers of diastolic function and left ventricular geometry. [file 2323410.f1.docx]

Supplemental Table 1. Associations of traditional risk factors and RA characteristics with markers of left ventricular diastolic function and left ventricular geometry

|  | Log E/A | | Log E/e' | | Log lateral e' | | Log septal e' | | LAVI | | RWT | | Log LVMI | |
| --- | --- | --- | --- | --- | --- | --- | --- | --- | --- | --- | --- | --- | --- | --- |
|  | partial r | p | partial r | p | partial r | p | partial r | p | partial r | p | partial r | p | partial r | p |
| Age | **-0.39** | **<0.001** | **0.19** | **0.01** | **-0.47** | **<0.001** | **-0.44** | **<0.001** | **0.28** | **0.0002** | 0.06 | 0.43 | **0.36** | **<0.001** |
| Sex | **0.16** | **0.03** | 0.11 | 0.15 | 0.07 | 0.37 | 0.05 | 0.49 | -0.07 | 0.33 | 0.15 | 0.06 | **-0.28** | **0.0002** |
| Body mass index | -0.07 | 0.40 | -0.02 | 0.79 | -0.04 | 0.63 | -0.13 | 0.10 | 0.09 | 0.28 | -0.12 | 0.14 | **0.22** | **0.007** |
| Waist | -0.14 | 0.07 | 0.03 | 0.67 | **-0.21** | **0.009** | -0.13 | 0.09 | **0.18** | **0.03** | -0.06 | 0.45 | **0.24** | **0.002** |
| Waist-hip ratio | **-0.24** | **0.003** | 0.04 | 0.59 | **-0.34** | **<0.001** | -0.14 | 0.08 | **0.27** | **0.0005** | 0.11 | 0.16 | **0.19** | **0.02** |
| Heart rate | **-0.29** | **0.0002** | -0.02 | 0.77 | -0.15 | 0.06 | -0.05 | 0.52 | -0.03 | 0.70 | 0.13 | 0.09 | -0.14 | 0.07 |
| Hypertension | -0.09 | 0.24 | -0.05 | 0.48 | -0.04 | 0.60 | -0.03 | 0.65 | **0.27** | **0.0003** | -0.04 | 0.65 | 0.12 | 0.11 |
| DBP | -0.03 | 0.67 | **-0.16** | **0.04** | **0.19** | **0.02** | -0.05 | 0.55 | -0.05 | 0.52 | -0.03 | 0.73 | 0.03 | 0.73 |
| HDL cholesterol | 0.13 | 0.11 | -0.12 | 0.12 | **0.18** | **0.02** | -0.01 | 0.89 | -0.10 | 0.21 | -0.07 | 0.37 | **-0.20** | **0.01** |
| Triglyderides* | -0.10 | 0.22 | -0.001 | 0.99 | **-0.20** | **0.01** | -0.001 | 0.99 | **0.25** | **0.001** | 0.01 | 0.86 | 0.05 | 0.57 |
| Dyslipidemia | -0.06 | 0.44 | 0.09 | 0.22 | **-0.20** | **0.009** | -0.06 | 0.46 | 0.10 | 0.18 | -0.03 | 0.66 | 0.12 | 0.11 |
| HOMA_IR* | **-0.17** | **0.04** | -0.04 | 0.60 | **-0.16** | **0.05** | -0.03 | 0.73 | **0.18** | **0.03** | -0.03 | 0.71 | 0.01 | 0.90 |
| Disease duration* | **-0.25** | **0.001** | 0.08 | 0.29 | **-0.28** | **0.0002** | **-0.35** | **<0.001** | **0.21** | **0.007** | 0.06 | 0.43 | **0.20** | **0.01** |
| ACPA | -0.11 | 0.16 | 0.13 | 0.09 | -0.01 | 0.92 | -0.01 | 0.88 | 0.06 | 0.43 | **0.18** | **0.02** | 0.08 | 0.29 |
| DAS28 | **-0.17** | **0.03** | -0.03 | 0.75 | -0.03 | 0.72 | -0.06 | 0.44 | -0.02 | 0.79 | 0.07 | 0.35 | -0.12 | 0.12 |
| ESR* | **-0.20** | **0.01** | -0.07 | 0.35 | 0.01 | 0.93 | -0.04 | 0.61 | 0.08 | 0.32 | 0.08 | 0.29 | -0.11 | 0.18 |
| C-reactive protein | **-0.16** | **0.04** | 0.01 | 0.90 | 0.03 | 0.69 | -0.10 | 0.20 | -0.01 | 0.89 | 0.06 | 0.45 | -0.07 | 0.40 |
| WCC* | 0.10 | 0.22 | 0.10 | 0.18 | 0.02 | 0.78 | 0.01 | 0.90 | 0.09 | 0.31 | **0.19** | **0.01** | -0.09 | 0.25 |
| EAM | -0.05 | 0.53 | 0.01 | 0.94 | -0.09 | 0.24 | -0.04 | 0.57 | **0.16** | **0.04** | -0.02 | 0.81 | -0.01 | 0.92 |
| Sulphasalazine | -0.01 | 0.89 | -0.01 | 0.87 | 0.009 | 0.90 | 0.08 | 0.32 | 0.01 | 0.89 | 0.02 | 0.80 | **0.18** | **0.02** |
| Azathioprine | -0.09 | 0.32 | 0.003 | 0.97 | 0.02 | 0.82 | **-0.17** | **0.02** | 0.01 | 0.87 | 0.01 | 0.87 | -0.09 | 0.27 |
| GFR* | **0.22** | **0.01** | -0.13 | 0.12 | **0.27** | **0.002** | **0.27** | **0.001** | **-0.17** | **0.04** | 0.001 | 0.99 | **-0.24** | **0.005** |

Data were analyzed in age, sex and race adjusted linear regression models; age was replaced by age at disease onset in the model on disease duration, and age, sex and race were omitted in the models on GFR. Significant associations are shown in bold. DBP, diastolic blood pressure; HDL, high density lipoprotein cholesterol; HOMA-IR, homeostasis model of insulin resistance; ACPA, anti-citrullinated peptide antibody; DAS28, disease activity score in 28 joints; ESR, erythrocyte sedimentation rate; WCC, white cell count; EAM, extra articular manifestations; GFR, glomerular filtration rate.

* Logarithmically transformed

Supplemental Table 2. Non-significant associations of traditional risk factors and RA characteristics with markers of diastolic function and left ventricular geometry

|  | Log E/A | | Log E/e' | | Log lateral e' | | Log septal e' | | LAVI | | RWT | | LVMI | |
| --- | --- | --- | --- | --- | --- | --- | --- | --- | --- | --- | --- | --- | --- | --- |
|  | partial r | p | partial r | p | partial r | p | partial r | p | partial r | p | partial r | p | partial r | p |
| Age dis onset | 0.02 | 0.77 | 0.07 | 0.36 | -0.001 | 0.99 | 0.02 | 0.76 | 0.002 | 0.97 | -0.10 | 0.19 | 0.10 | 0.20 |
| Race | 0.02 | 0.98 | 0.08 | 0.32 | -0.04 | 0.56 | -0.07 | 0.34 | -0.14 | 0.07 | 0.09 | 0.20 | -0.02 | 0.85 |
| Exercise | -0.01 | 0.86 | 0.08 | 0.32 | 0.02 | 0.81 | -0.11 | 0.14 | -0.04 | 0.64 | -0.05 | 0.49 | 0.02 | 0.81 |
| Alcohol | -0.06 | 0.45 | -0.05 | 0.53 | 0.07 | 0.36 | -0.12 | 0.12 | 0.29 | 0.70 | 0.01 | 0.88 | -0.01 | 0.88 |
| SBP | 0.002 | 0.98 | -0.03 | 0.67 | 0.11 | 0.16 | -0.003 | 0.97 | 0.08 | 0.27 | -0.03 | 0.70 | -0.03 | 0.66 |
| Pulse pressure | 0.03 | 0.75 | 0.07 | 0.35 | 0.00 | 0.99 | 0.03 | 0.71 | 0.14 | 0.08 | -0.02 | 0.83 | -0.05 | 0.45 |
| Total cholesterol | 0.06 | 0.46 | -0.13 | 0.1 | 0.07 | 0.40 | -0.01 | 0.94 | 0.03 | 0.71 | -0.04 | 0.64 | -0.13 | 0.10 |
| LDL cholesterol | 0.06 | 0.43 | -0.09 | 0.26 | 0.06 | 0.45 | 0.05 | 0.50 | 0.01 | 0.88 | -0.01 | 0.95 | -0.05 | 0.56 |
| Chol/HDL chol* | -0.09 | 0.28 | 0.03 | 0.66 | -0.15 | 0.06 | -0.01 | 0.93 | 0.15 | 0.05 | 0.05 | 0.56 | 0.07 | 0.39 |
| Diabetes | -0.05 | 0.57 | -0.03 | 0.73 | 0.01 | 0.91 | -0.13 | 0.11 | 0.02 | 0.77 | 0.01 | 0.91 | -0.08 | 0.28 |
| Glucose* | -0.07 | 0.36 | -0.04 | 0.64 | -0.06 | 0.47 | -0.06 | 0.48 | 0.12 | 0.14 | -0.10 | 0.23 | -0.02 | 0.76 |
| OGLA | -0.11 | 0.16 | -0.08 | 0.34 | -0.002 | 0.98 | -0.12 | 0.13 | -0.05 | 0.54 | -0.03 | 0.75 | -0.13 | 0.10 |
| Insulin treatment | -0.03 | 0.68 | -0.09 | 0.28 | -0.02 | 0.76 | -0.04 | 0.62 | 0.04 | 0.61 | -0.03 | 0.76 | 0.03 | 0.75 |
| RF positive | -0.13 | 0.11 | 0.06 | 0.47 | -0.001 | 0.99 | -0.001 | 0.99 | -0.02 | 0.83 | 0.03 | 0.71 | 0.06 | 0.41 |
| CDAI* | -0.02 | 0.76 | 0.002 | 0.98 | -0.06 | 0.41 | -0.02 | 0.54 | -0.03 | 0.72 | 0.05 | 0.49 | -0.04 | 0.65 |
| SDAI* | -0.03 | 0.72 | -0.05 | 0.55 | -0.02 | 0.83 | 0.02 | 0.80 | -0.08 | 0.33 | 0.11 | 0.14 | -0.04 | 0.60 |
| DAS28-CRP | -0.13 | 0.09 | 0.02 | 0.85 | -0.02 | 0.78 | -0.08 | 0.30 | -0.07 | 0.38 | 0.06 | 0.46 | -0.10 | 0.19 |
| Deformed joint* | -0.02 | 0.84 | 0.09 | 0.23 | -0.13 | 0.10 | -0.003 | 0.94 | -0.01 | 0.95 | 0.12 | 0.12 | -0.03 | 0.66 |
| Methotrexate | -0.03 | 0.71 | -0.03 | 0.69 | 0.03 | 0.69 | 0.02 | 0.78 | 0.07 | 0.36 | -0.03 | 0.76 | 0.14 | 0.08 |
| Chloroquine | -0.05 | 0.56 | 0.01 | 0.92 | 0.06 | 0.47 | 0.03 | 0.73 | -0.05 | 0.53 | -0.01 | 0.87 | 0.001 | 0.99 |
| Leflunomide | -0.06 | 0.47 | 0.26 | 0.74 | -0.10 | 0.19 | -0.08 | 0.32 | -0.01 | 0.93 | 0.03 | 0.65 | 0.01 | 0.91 |
| Tetracycline | -0.08 | 0.34 | -0.07 | 0.38 | -0.03 | 0.65 | 0.03 | 0.73 | 0.02 | 0.78 | 0.01 | 0.89 | 0.01 | 0.91 |
| Biologic agent use | 0.15 | 0.07 | -0.003 | 0.97 | -0.05 | 0.48 | 0.02 | 0.82 | 0.01 | 0.93 | -0.004 | 0.96 | -0.02 | 0.83 |
| TNF-α | 0.08 | 0.33 | -0.04 | 0.59 | -0.04 | 0.60 | 0.01 | 0.85 | -0.05 | 0.54 | -0.08 | 0.28 | 0.03 | 0.72 |
| Abatacept | 0.16 | 0.06 | 0.07 | 0.36 | -0.04 | 0.62 | 0.01 | 0.89 | 0.10 | 0.19 | 0.15 | 0.06 | -0.09 | 0.27 |
| NSAID | 0.10 | 0.23 | 0.02 | 0.81 | 0.05 | 0.50 | -0.07 | 0.35 | -0.003 | 0.97 | -0.03 | 0.69 | -0.10 | 0.18 |
| Prednisone | 0.06 | 0.48 | -0.03 | 0.67 | 0.08 | 0.29 | 0.11 | 0.16 | -0.002 | 0.98 | 0.14 | 0.06 | -0.01 | 0.86 |

Data were analyzed in age, sex and race adjusted linear regression models. Dis, disease; SBP, systolic blood pressure; LDL, low density lipoprotein cholesterol; HDL, high density lipoprotein; chol, cholesterol; OGLA, oral glucose lowering agents; RF, rheumatoid factor; CDAI, clinical disease activity index; SDAI, simple disease activity index; DAS28, disease activity score in 28 joints; TNFα, tumor necrosis factor alpha inhibitors; NSAID, non-steroidal anti-inflammatory drugs.

* Logarithmically transformed.
